# Biophysical Regulation of Chromatin Architecture Instills a Mechanical Memory in Mesenchymal Stem Cells

**DOI:** 10.1038/srep16895

**Published:** 2015-11-23

**Authors:** Su-Jin Heo, Stephen D. Thorpe, Tristan P. Driscoll, Randall L. Duncan, David A. Lee, Robert L. Mauck

**Affiliations:** 1 McKay Orthopaedic Research Laboratory, Department of Orthopaedic Surgery, Perelman School of Medicine, University of Pennsylvania, Philadelphia, PA, USA; 2 Department of Bioengineering, School of Engineering and Applied Science, University of Pennsylvania, Philadelphia, PA, USA; 3 Institute of Bioengineering, School of Engineering and Materials Science, Queen Mary University of London, London, UK; 4 Translational Musculoskeletal Research Center, Philadelphia VA Medical Center, Philadelphia, PA, USA; 5 Department of Biological Sciences, University of Delaware, Newark, DE, USA

## Abstract

Mechanical cues direct the lineage commitment of mesenchymal stem cells (MSCs). In this study, we identified the operative molecular mechanisms through which dynamic tensile loading (DL) regulates changes in chromatin organization and nuclear mechanics in MSCs. Our data show that, in the absence of exogenous differentiation factors, short term DL elicits a rapid increase in chromatin condensation, mediated by acto-myosin based cellular contractility and the activity of the histone-lysine N-methyltransferase EZH2. The resulting change in chromatin condensation stiffened the MSC nucleus, making it less deformable when stretch was applied to the cell. We also identified stretch induced ATP release and purinergic calcium signaling as a central mediator of this chromatin condensation process. Further, we showed that DL, through differential stabilization of the condensed chromatin state, established a ‘mechanical memory’ in these cells. That is, increasing strain levels and number of loading events led to a greater degree of chromatin condensation that persisted for longer periods of time after the cessation of loading. These data indicate that, with mechanical perturbation, MSCs develop a mechanical memory encoded in structural changes in the nucleus which may sensitize them to future mechanical loading events and define the trajectory and persistence of their lineage specification.

Mesenchymal stem cells (MSCs) are a promising cell source for regenerative therapies given their multipotent nature^1^,^2^. These cells are exquisitely sensitive not just to soluble differentiation factors, but also to biophysical cues arising from or induced by the cellular microenvironment, including substrate stiffness^3^, cell morphology^3^,^4^, and dynamic mechanical perturbation^5^,^6^. These exogenous mechanical cues influence cytoskeletal organization, cell proliferation and differentiation, chromatin remodeling and nuclear stiffness, and ultimately the genetic program that defines lineage specification^7^,^8^,^9^,^10^.

The nucleus is the largest and stiffest organelle of a mammalian cell, housing the majority of its genetic material and serving as a focal point for mechanotransduction via its attachments to the cytoskeletal network^11^,^12^. For instance, we recently showed that nuclear deformation mediated by nuclear connectivity through the large LINC complex member Nesprin 1 Giant was essential for nuclear YAP/TAZ signaling in response to stretch^11^. In addition to these connectivity-mediated signaling events, structural elements within the nucleus, including chromatin and the proteinaceous components of the nuclear lamina, determine the transcriptional activity of the cell and define nuclear stiffness, both of which change during differentiation^13^,^14^,^15^,^16^. In contrast to euchromatin, the condensed chromatin state (heterochromatin) is associated with gene silencing^17^,^18^. Indeed, differentiation is typified by chromatin condensation, leading to an overall gene silencing while preserving lineage-specific gene expression in small euchromatic niches^19^,^20^. Chromatin condensation is mediated by histone methyl- and acetyl-transferases, de-methylases and de-acetylases^21^ that coordinately regulate the epigenetic landscape local to gene sets that define a lineage.

Mechanical perturbations can alter the state of the nucleus, with some suggesting that physical signals reach the nucleus more rapidly than soluble ones, enabling more efficient conveyance of mechanical information to the genome^22^. For example, seminal work by Deguchi and colleagues showed that fluid flow induced shear stress modulates chromatin condensation and increases nuclear stiffness in endothelial cells^8^. Likewise, direct force transmission to the nucleus through the cytoskeleton (via magnetic bead twisting on the apical surface) elicits local chromatin remodeling within seconds^9^. Even in isolated nuclei, stretch applied through LINC complex coated beads results in remodeling of the nuclear lamina and stiffening of the nucleus, within just a few cycles of mechanical perturbation^23^.

Despite this growing appreciation of the role for mechanical stimuli in guiding lineage specification and regulating genome architecture, the molecular machinery through which these perturbations culminate in chromatin remodeling has not yet been fully elucidated. In this study, we established strain magnitudes and timing over which dynamic tensile loading (DL) altered chromatin remodeling, and identified the primary molecular mechanisms governing this process, with a specific focus on stretch induced ATP release and subsequent purinergic and stretch-activated channel mediated calcium signaling^24^,^25^,^26^,^27^. Further, as some loading configurations and molecular pathways resulted in persistent changes in chromatin, we explored how loading might establish a ‘mechanical memory’ in these cells^28^,^29^, via the persistence of load-induced alterations in their chromatin architecture.

## Results

### Rapid Alteration of MSC Chromatin Condensation in Response to Dynamic Stretch

**Figure 1:**
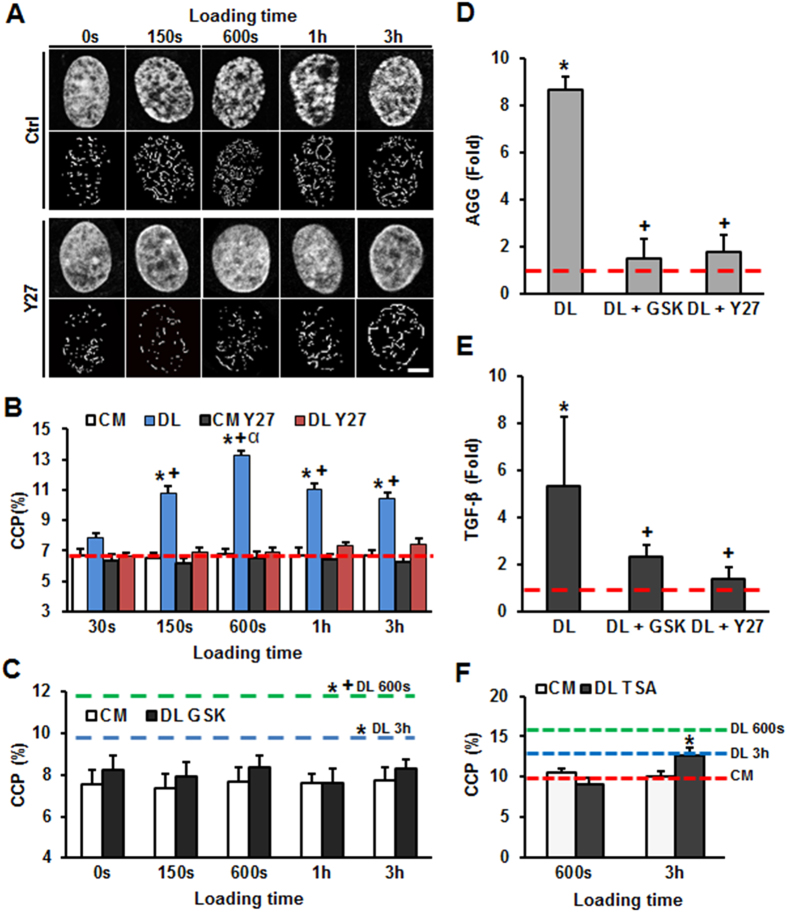
Dynamic loading (DL) is transmitted through the contractile cytoskeleton to induce rapid chromatin condensation and alter gene expression in MSCs via the activity of histone modifying enzymes. (**A**) DL (3%, 1 Hz) for 150 seconds, 600 seconds, 1 hour, and 3 hours in the absence of exogenous growth factors increases apparent chromatin condensation in DAPI stained nuclei (top row), and increases the number of visible edges within nuclei after CCP image processing (bottom row) (Ctrl). Little change in chromatin structure was observed with DL when MSCs were pre-treated with a Rho-kinase inhibitor before DL (DL Y27, bar = 3 μm). (**B**) Quantification of the chromatin condensation parameter (CCP) in response to DL and with inhibition of acto-myosin contractility with Y27 treatment (red line: CCP of unloaded MSCs in control media (CM) at 0 sec, n = ~40 cells per condition per time point, from 2 replicate studies, *p < 0.05 vs. CM control, ^+^p < 0.05 vs. Y27, ^α^p < 0.05 vs. 150s). (**C**) CCP with loading in the context of blockade of Enhancer of Zeste Homolog 2 (EZH2) activity by GSK343 treatment (GSK, green line: CCP of DL MSCs at 600s, blue line: CCP of DL MSCs at 3h, n = ~40 cells per condition per time point, from 2 replicate studies, *p < 0.05 vs. CM at 0 sec, ^+^p < 0.05 vs. DL 3 h in CM, mean ± s.e.m.). DL (3%, 1 hz, 3 h) increased aggrecan (AGG, **D**) and TGF-β (**E**) gene expression in the absence of growth factors, but showed no change with either GSK or Y27 pre-treatment (fold change relative to 0% strain control and normalized to GAPDH, n = 3, *p < 0.05 vs. 0% control, ^+^p < 0.05 vs. DL, mean ± s.d.). (**F**) Inhibition of histone deacetylases by pre-treatment with trichostatin A (TSA) abrogated load induced chromatin condensation with 600s DL, but not with 3 hr DL (red line: CCP of control MSCs, green line: CCP of DL MSCs at 600s, blue line: CCP of DL MSCs at 3h, n = 20 cells per condition per time point, *p < 0.05 vs. CM control, mean ± s.e.m.).

Naïve mesenchymal stem cells (MSCs) were seeded onto aligned nanofibrous scaffolds and subjected to dynamic tensile loading (DL). In the absence of exogenous differentiation factors, 3% strain applied at 1 Hz resulted in marked chromatin condensation, as was evidenced by the appearance of prominent edges in DAPI stained nuclei. This increase in condensation was evident after 150 seconds, reaching peak values after 600 seconds of DL (Fig. 1A,B). Quantification of this edge densification through the computation of a ‘chromatin condensation parameter’ (CCP)^30^, showed a nearly 100% increase in nuclear edge density at this time point. Longer term DL (for 1 and 3 hours) also showed increases in CCP of 50–75% compared to unloaded controls. This pattern of CCP reaching higher values early (at 600 sec) compared to later (at 3 hours) was consistent over multiple experiments. While the mechanism for this is not clear, it was evident that when cell contractility was eliminated through Rho-associated protein kinase (ROCK) inhibition (with Y27632), chromatin condensation did not change with loading, across all time points (Fig. 1A,B). Likewise, expression of aggrecan (AGG) and TGF-β increased with 3 hours of loading, and this change was blocked by the addition of Y27362 (Fig. 1D,E). Consistent with previous findings^9^, this data supports the notion that a patent acto-myosin contractility apparatus is required for chromatin remodeling and regulation of load induced changes in gene expression.

To determine whether the condensation observed was simply a physicochemical phenomenon (e.g., increased DNA packing density mediated by changes in osmolarity of intracellular compartments with loading^30^) or an actual biologic pathway regulating chromatin organization, we next probed the role of enzymes associated with histone modification on this process. Specifically, we inhibited the histone-lysine N-methyltransferase Enhancer of Zeste Homolog 2 (EZH2), an enzyme that catalyzes the addition of methyl groups to histone H3^31^, using the small molecule inhibitor GSK343^32^. Addition of GSK343 eliminated load-induced changes in CCP across all time points, with little change in chromatin condensation observed after either 600 seconds or 3 hours of DL in the presence of inhibitor (Fig. 1C), along with similar inhibition of load-induced changes of AGG and TGF-β gene expression (Fig. 1D,E). Interestingly, when histone deacetylases (HDACs) were inhibited via the addition of Trichostatin A (TSA), DL induced chromatin condensation was only blocked with short (600 seconds), but not longer term (3 hour) loading (Fig. 1F). These data suggest that DL induced chromatin condensation requires activation of methyl-transferases to mediate chromatin remodeling in the short and long term, while deacetylases were only required in the short term.

### Persistence of Load-Induced Chromatin Condensation and its Impact on Nuclear Mechanics

**Figure 2:**
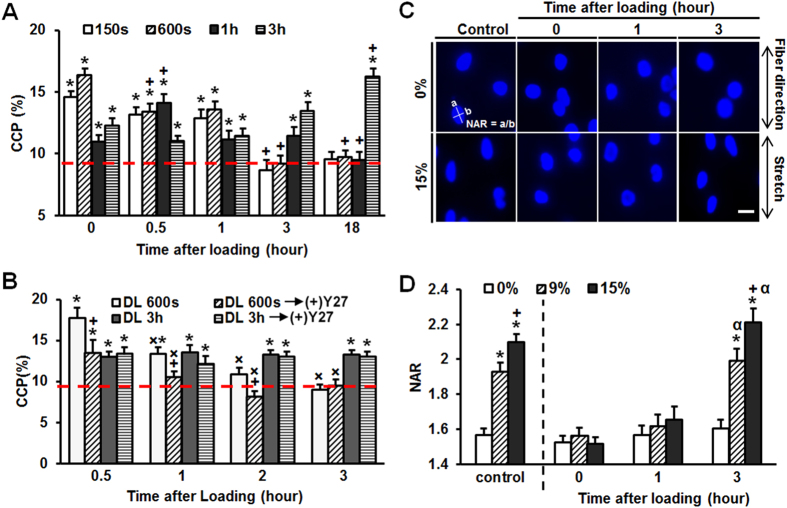
Persistence of chromatin condensation depends on the duration of stimulation and alters nuclear mechanics. (**A**) Chromatin condensation (CCP) gradually decreased to baseline levels within 3 hours of the cessation of 150s or 600s of DL. The rate of CCP relaxation was slower after 1 h of DL, and did not decrease over an 18 hour observation window following 3 hr of DL (red line: unloaded CM control, n = 20, *p < 0.05 vs. CM control, ^+^p < 0.05 vs. 0 h, mean ± s.e.m.). (**B**) Persistency in condensation after 600 s (but not 3 hr) of DL depended on acto-myosin contractility (red line: unloaded CM control, n = 20, *p < 0.05 vs. CM control, ^+^p < 0.05 vs. without Y27 treatment, ^×^p < 0.05 vs. 0.5 h mean ± s.e.m.). (**C**) representative DAPI stained nuclei on scaffolds post 600 s DL with/without 15% static stretch (bar = 10 μm), (**D**) Nuclear aspect ratio (NAR) with 9% and 15% applied stretch (n = ~40 cells, *p < 0.05 vs. 0%, ^+^p < 0.05 vs. 9%, ^α^p < 0.05 vs. 0 hour for same strain level, mean ± s.e.m.).

Having observed a rapid condensation of chromatin in response to loading, we interrogated how long these changes persist after loading ceased, and whether the degree of permanency depended on the duration or magnitude of loading. To answer these questions, samples were cultured for 18 hours following loading bouts of 150 s, 600 s, 1 hr, or 3 hrs. Results showed that the increased chromatin condensation persisted for different amounts of time, depending on the duration of the original stimulation. After short term DL (150s and 600s), CCP progressively decreased, reaching baseline levels within 3 hours of loading cessation. Conversely, when DL was applied for 1 hour, relaxation of the chromatin condensation was more gradual, not reaching control levels until between 3 and 18 hours (Fig. 2A). With 3 hours of DL, on the other hand, chromatin condensation remained elevated, and even slightly increased, over the 18-hour observation window (Fig. 2A).

In addition to the duration of DL, the rate of chromatin relaxation also depended on the amplitude of applied dynamic strain. That is, CCP reached higher levels and remained elevated for a longer duration when samples were dynamically loaded to 9% strain compared to 3% strain (sFig. 1). Interestingly, acto-myosin contractility influenced the rate of chromatin relaxation after short term loading (600s), but not long term loading (3h) (Fig. 2B). These data indicate that architectural changes in the nucleus are not simply the result of an increase in overall cell contractility in response to load, but rather depend on physical changes within the nucleus, and that there may be distinct mechanisms controlling early versus longer term chromatin condensation in response to loading.

To determine if the observed changes in chromatin condensation influenced nuclear deformability, we applied graded levels of static stretch to the whole cell and visualized nuclear deformation at various time points after cessation of dynamic loading. Nuclei in cells pre-conditioned with 600 seconds of DL showed attenuated deformation compared to control MSCs immediately after the cessation of loading (Fig. 2C,D). When the same test was performed 3 hours after the cessation of loading, nuclear deformability had returned to baseline levels. This suggests that DL rapidly alters chromatin structure, resulting in an increase in nuclear stiffness, which dissipates with increasing time after loading as the condensed chromatin relaxes towards its baseline configuration. This relationship between nuclear stiffness and chromatin condensation was confirmed by an alternative method to temporarily condense the chromatin, namely the transient application of hyperosmotic shock^30^. This treatment rapidly increased chromatin condensation, produced nuclei that were resistant to deformation, and increased the peri-nuclear stiffness (sFig. 2). Collectively, these data suggest that alterations in chromatin condensation mediated by both dynamic loading (and other methods that regulate chromatin condensation) result in a nucleus that is stiffer and more resistant to subsequent deformation, potentially sensitizing the cell to further mechano-response.

### Mechanically Induced Purinergic Signaling Initiates Chromatin Condensation

**Figure 3:**
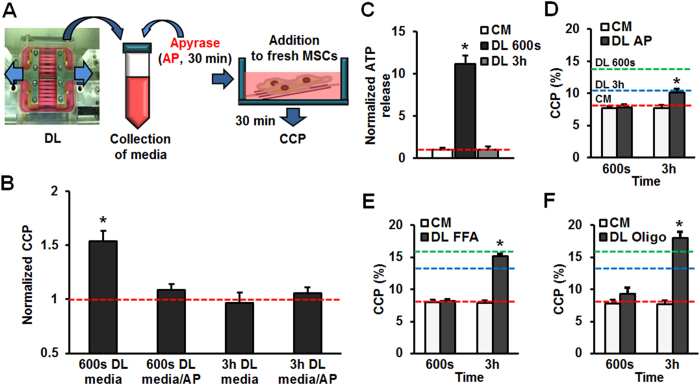
ATP release and purinergic signaling are mediators of loading-induced chromatin condensation in the short but not the long term. (**A**) Schematic illustration of conditioned media studies including treatment with apyrase (AP). (**B**) Normalized CCP values (relative to unloaded CM MSCs) after treatment for 30 minutes with DL-conditioned media (n = ~20, *p < 0.05 vs. CM control (red line), mean ± s.e.m.). (**C**) ATP concentration in the media increases after DL for 600s, but not after DL for 3h (normalized to CM, n = 3, *p < 0.05, mean ± s.d.). (**D–F**) Pharmacologic inhibition of ATP signaling abrogated the short term (600 s) loading induced changes in CCP, but not did not alter the response to long term (3 h) loading (green line: CCP value with 600s DL, blue line: CCP value with 3 h DL, red line: CCP in control MSCs in CM, n = ~20, *p < 0.05 vs CM control, AP: apyrase, FFA: flufenamic acid, Oligo: oligomycin, mean ± s.e.m.).

Having established that dynamic loading alters the chromatin state, we next sought to determine the mechanism by which this occurs, and in particular if soluble factors released upon loading initiate signaling. To do so, MSCs on scaffolds were exposed to DL for 600 seconds, after which the medium was collected. A portion of this DL-conditioned media was added to fresh scaffolds, and chromatin condensation (CCP) was assessed (Fig. 3A). Results showed that addition of DL-conditioned media to fresh MSC-seeded scaffolds increased their chromatin condensation (CCP) to levels matching that of dynamically loaded cells (Fig. 3B). To determine the size of the effector molecule, load-conditioned media was size fractionated. Results showed that when the <3 kDa DL-conditioned fraction was added to fresh MSC-seeded scaffolds, a ~30% increase in CCP was observed, while addition of DL-conditioned medium containing the >3 kDa fraction had no effect on CCP (sFig. 3). This suggested that the effector molecules in DL-conditioned media were small, and so we first focused our attention on ATP (~507 Da) as a potential mediator of chromatin condensation as this molecule is a central player in early signaling in response to mechanical perturbation, and purinergic signaling plays important roles in stem cell differentiation^25^,^27^. To validate that the effector was ATP, DL-conditioned medium was treated with Apyrase (AP), an exogenous ATP diphosphohydrolase. When added to fresh scaffolds for 30 min, AP treated DL-conditioned medium did not elicit a change in CCP (Fig. 3B). Conversely, the addition of exogenous ATP to fresh scaffolds induced a dose dependent response, with chromatin condensation increasing with ATP concentration up to 0.5 mM (sFig. 4A). Similarly, treatment with the P2 receptor agonist UTP increased chromatin condensation, while BzATP, an agonist of the P2X receptors, did not alter chromatin condensation (sFig. 4B). This suggests that ATP signaling through the G protein-coupled receptor P2Y2 is responsible for DL-induced chromatin condensation in MSCs.

Given that changes in chromatin condensation were dependent on dynamic loading duration, and that ATP release was required for this process, we next probed whether soluble factors released within specific time frames were sufficient to drive chromatin condensation. Interestingly, when we added the conditioned media collected from constructs after 3 hours of mechanical loading, no change in CCP was observed and addition of AP had no effect on the resultant CCP in recipient cells (Fig. 3B). By directly measuring ATP in the DL-conditioned medium, we found a 12-fold increase in ATP concentration after 600 seconds of loading, but little change in ATP in media collected after 3 hours of DL (Fig. 3C). Given that ATP can be broken down quickly, we measured ATP in the media as a function of time, in the absence and presence of cells. These assays showed that 60% of ATP released with 600 seconds of DL was hydrolyzed after 3 hours in the absence of cells, and that this rate of hydrolysis/consumption increased when cultured with unloaded MSCs (sFig. 5). Interestingly, when we continuously hydrolyzed released extracellular ATP via AP treatment during loading, we found no change in CCP over the short term (600 seconds), but still saw a modest increase at 3 hours (Fig. 3D). Similar results (early blockade but later increases in CCP) were observed when we treated with either flufenamic acid (FFA: a hemichannel blocker, Fig. 3E) or oligomycin (Oligo: an inhibitor of ATP synthesis, Fig. 3F). These data suggest that the short term response to DL is primarily mediated by purinergic signaling, but that other pathways are activated over the longer term. Both, however, depend ultimately on the action of EZH2 and cell contractility, as inhibition of these factors blocked both the short and long term changes in CCP with DL.

We also found that the transcriptional regulator YAP (Yes-associated protein), which relays mechanical signals via its translocation to the nucleus^11^, was present in the cytoplasm of MSCs on scaffolds in unloaded conditions, while treatment with either 1mM of ATP or application of DL for 30 minutes resulted in YAP mobilization to the nucleus (sFig. 6A–D). Blockade of purinergic signaling by addition of Apyrase or FFA during short term DL inhibited the nuclear localization of YAP (sFig. 6E). This suggests that load-induced purinergic signaling may play a role in this process as well, perhaps through alterations in cell contractility^33^.

### Calcium Signaling is Required for Load Induced Chromatin Condensation

**Figure 4:**
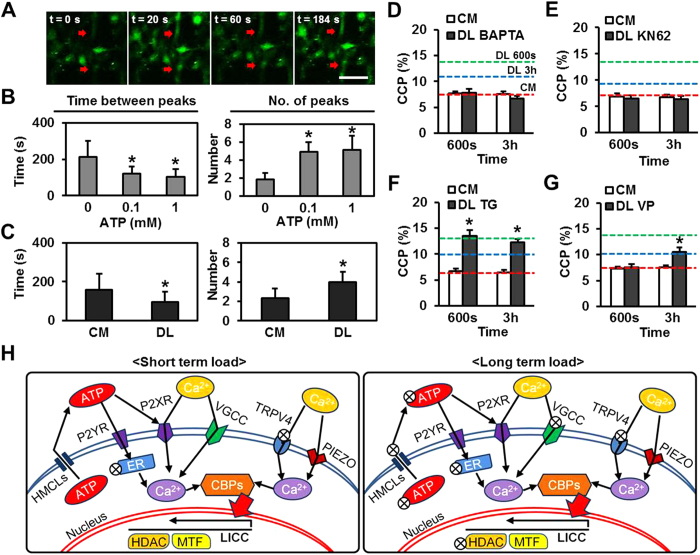
Loading induced chromatin condensation is regulated by calcium signaling. (**A**) Representative [Ca^2+^]_i_ oscillations (red arrows) in MSCs as a function of time (bar = 100 μm). Addition of ATP (**B**) or application of 30s DL (**C**) decreased the time between peeks and increased number of peaks observed in 10 min (n = ~15, *p < 0.05 vs. CM control (0% strain/0mM ATP, mean ± s.d.). (**D,E**) Pretreatment with BAPTA or KN62 blocked load-induced chromatin condensation, whereas pretreatment with thapsigargin (TG, **F**) had no effect and Verapamil (VP, **G**) blocked only the short term increase in CCP (at 600s of DL) (red line: CM control, green line: 600s DL, blue line: 3 h DL, n = ~20 per condition, *p < 0.05 vs. CM control, mean ± s.e.m.). (**H**) Schematic illustration outlining the operative signaling pathways controlling chromatin condensation with short term (600s) or long term (3h) loading (⊗ a component that is not on the critical path for load induced chromatin condensation at that time point. ATP: Adenosine triphosphate, HMCLs: hemichannels, P2YR: P2Y purinergic receptors, P2XR: P2X purinergic receptors, ER: endoplasmic reticulum, Ca: calcium. CBPs: calcium binding proteins, VGCC: voltage-gated calcium channels, TRPV4: Transient receptor potential cation channel subfamily V member 4, PIEZO: Piezo-type mechanosensitive ion channels, HDAC: Histone deacetylase, MTF: Histone methyltransferase, LICC: load induced chromatin condensation).

Given that dynamic loading caused ATP release, in turn activating purinergic signaling, which is usually mediated through calcium entry, we next probed the role of downstream calcium signaling in DL-induced chromatin condensation. For this, MSCs on scaffolds were stained with Cal-520^TM^, and calcium signaling was monitored before and after loading. At rest, unloaded MSCs showed regular [Ca^2+^]_i_ oscillations over a 10 min observation period (Fig. 4A). The addition of exogenous ATP increased the frequency and number of these Ca^2+^ oscillations (Fig. 4B). Dynamic loading for 600 seconds had a similar effect, decreasing the time between peaks and increasing the number of peaks in the 10 min observation window after loading (Fig. 4C).

Based on these results, we next used pharmacologic inhibitors to manipulate calcium signaling. Extracellular Ca^2+^ ions played crucial roles in DL-induced chromatin condensation; the addition of the calcium chelators BAPTA or EGTA to the culture media completely blocked chromatin condensation at both short (600 seconds) and long (3 hours) loading durations (Fig. 4D and sFig. 7A). Likewise, when antagonists to downstream calcium binding proteins [KN-62 (KN): a Calmodulin kinase II inhibitor, CALP2: an antagonist of Calmodulin, Cyclosporine A (CYSP): a Calcineurin inhibitor] were added, DL for both the short and long durations failed to alter CCP (Fig. 4E and sFig. 7B,C). Conversely, addition of BAPTA-AM (BATAM) to sequester calcium released from intracellular stores only blocked chromatin condensation in response to short term loading, while thapsigargin (TG) had no effect at either time point (Fig. 4F and sFig. 7D). This suggests that intracellular Ca^2+^ pools have a lesser role in DL-induced chromatin condensation^34^. Verapamil (VP, a voltage-gated calcium channel blocker) also blocked DL-induced chromatin condensation only in response to short term (but not long term) loading (Fig. 4G). TRPV4 was not involved in load induced chromatin condensation at either time point, as verified by pre-treatment with Ruthenium Red (sFig. 7E) and the addition of the TRPV4 antagonist, GSK205 (G205, sFig. 7F). However, loading induced chromatin condensation was completely abolished by blocking the PIEZO family of ion channels through the addition of GsMTx4 (GMT, sFig. 7G). When MSCs were pretreated with GdCl_3_ (GC) to block stretch-activated channels or PPADS (a P2 receptor antagonist), chromatin condensation in response to DL was blocked in both the short and long term (sFig. 7H,I). Control experiments were carried out to ensure that addition of these pharmacologic inhibitors did not alter baseline CCP levels (sFig. 8A) or nuclear deformability (sFig. 8B).

In summary, ATP/purinergic signaling was essential for the short term DL-induced chromatin condensation response, while calcium signaling was critical for both short and long term responses (Fig. 4H). Voltage-gated calcium channels (VGCC) and HDACs were only involved in DL-induced chromatin condensation in the short term, while PIEZO channels and histone methyltransferases (MTFs) were important for both the short and long term response (Fig. 4H). TRPV4 channels and intracellular Ca^2+^ stores were generally not involved in loading induced chromatin condensation (Fig. 4H).

### Loading Instills a ‘Mechanical Memory’ in MSC Nuclei

**Figure 5:**
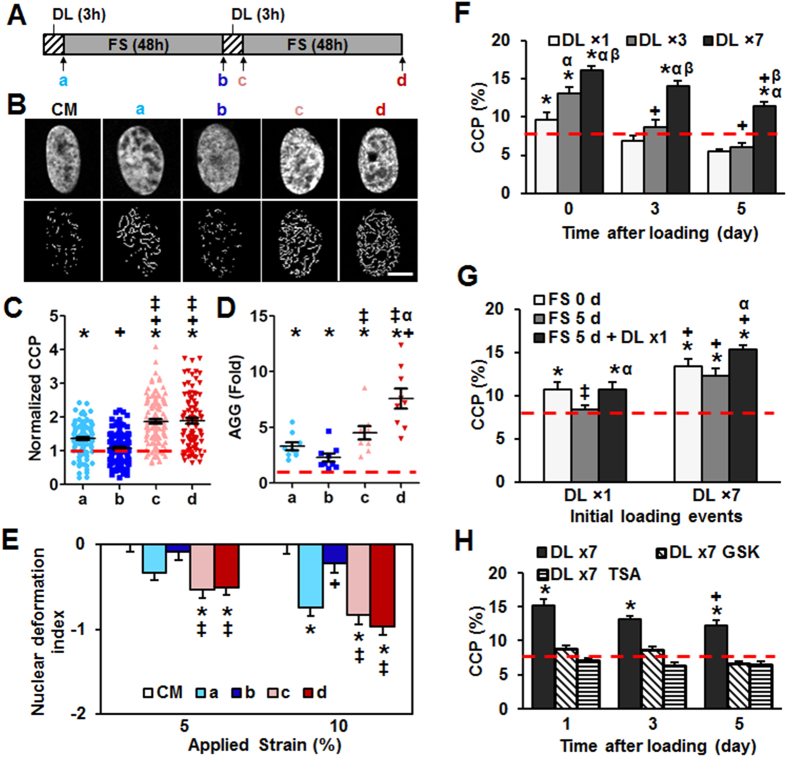
Loading history establishes a mechanical memory encoded in chromatin condensation. (**A**) Schematic showing experimental setup (DL: dynamic loading, FS: free swelling, a: immediately after 1^st^ 3h loading, b: 48 hours after the 1^st^ loading, c: immediately after 2^nd^ 3h loading, d: 48 hours after the 2^nd^ loading). (**B**) Representative DAPI stained nuclei (top row) and corresponding detection of visible edges (bottom row) for time points indicated in **(A)**, bar = 3 μm. (**C**) CCP normalized to CM control MSCs (red line: CM control, n = ~120 from 4 replicates, *p < 0.05 vs. CM control, ^+^p < 0.05 vs. a, ^‡^p < 0.05 vs. b, mean ± s.e.m.). (**D**) AGG gene expression normalized to CM control (n = 9, from 3 replicates, *p < 0.05 vs. CM control, ^+^p < 0.05 vs. a, ^‡^p < 0.05 vs. b, ^α^p < 0.05 vs. c, mean ± s.e.m.). (**E**) Nuclear deformation index (NDI) with multiple loading cycles, n = ~ 50, *p < 0.05 vs. CM control, ^+^p < 0.05 vs. a, ^‡^p < 0.05 vs. b, mean ± s.e.m.). (**F**) Alterations in CCP as a function of initial DL events and time after loading (n = ~20, *p < 0.05 vs. CM control (red line), ^+^p < 0.05 vs. day 0, ^α^p < 0.05 vs. DL × 1, ^β^p < 0.05 vs. DL × 3, mean ± s.e.m.). (**G**) Alterations in CCP with an additional loading event applied 5 days after cessation of initial DL events (n = ~20, *p < 0.05 vs. CM control (red line), ^+^p < 0.05 vs. DL × 1, ^‡^p < 0.05 vs. FS 0 d, ^α^p < 0.05 vs. FS 5 d, mean ± s.e.m.). (**H**) Alterations in CCP with inhibition of histone deacetylases and methyltransferases after seven days of loading (TSA: trichostatin A and GSK: GSK343 respectively, n = ~20, *p < 0.05 vs. CM control (red line), ^+^p < 0.05 vs. day 1, mean ± s.e.m.).

Given that both chromatin relaxation studies and pathway analysis identified differential responses depending on the duration of loading, we next queried whether and how previous loading events might be ‘imprinted’ on the nucleus after the cessation of mechanical loading, and whether multiple loading events alter the permanency of these changes in nuclear architecture. MSC-seeded scaffolds were loaded for 3 hours (a), maintained in unloaded culture for 48 hours (b), loaded again for 3 hours (c), and then maintained in unloaded culture for a further 48 hours (d) (Fig. 5A). Results from this study showed that chromatin condensation relaxed to an equilibrium state after 48 hours of unloaded (free swelling) culture following the first loading event, but that after a second loading event, higher levels of condensation were achieved and these were sustained over the subsequent 48 hours (Fig. 5B,C). Similarly, the 2nd loading event caused a much greater increase in AGG gene expression than the 1st loading event, and this higher level of expression increased in the 48 hours following the 2nd loading, while it decreased back to baseline in the 48 hours after the 1st loading (Fig. 5D). Expression of TGF-β exhibited a similar pattern (sFig. 9A). In addition, SMC1A, a subunit of the cohesion complex that is involved in chromatin mobility, was up-regulated with both loading events and increased more during rest periods (sFig. 9B). Likewise, CTCF, a transcriptional regulator that binds to chromatin insulators, was also up-regulated by both loading events and remained highly expressed during the 2^nd^ rest period (sFig. 9C). Consistent with previous findings relating chromatin condensation to nuclear deformability, a single bout of loading decreased nuclear deformability (negative nuclear deformation index indicates less deformation than control), which returned to baseline levels after 48 hours. Conversely, two bouts of loading resulted in a decrease in nuclear deformability that remained lower than control nuclei through an additional 48 hours (Fig 5E). Collectively, these data support the notion that repeated loading events instill a mechanical memory in the chromatin architecture, nuclear mechanics, and the expression profile of dynamically loaded MSCs.

As a final study, we asked how long might these changes be imprinted and whether the number of loading cycles mattered. To answer this question, MSC-seeded scaffolds were loaded one time (×1), 3 times (×3), or 7 times (×7) in daily 3 hour DL events, and chromatin condensation was monitored over the following five days. These studies showed that the initial number of loading cycles influenced both the magnitude of chromatin condensation achieved and its permanency after cessation of loading (Fig. 5F). In particular, the CCP did not return to baseline levels following 7 loading events, even after 5 days of unloaded culture. Aggrecan (AGG) expression followed this same general trend (sFig. 10). To determine whether a past condensation influences a future condensation, another set of constructs were dynamically loaded for either 1 day (DL × 1) or 7 days (DL × 7), cultured unloaded for an additional 5 days to allow for relaxation (if it was to occur), and then subjected to another round of loading. CCP values decreased to baseline levels 5 days after 1 loading bout (×1), and increased to the same level with another loading bout. With 7 prior bouts of loading (×7) however, there was no difference in the CCP immediately following loading and after 5 days of unloaded culture, and a small increase was observed from this higher baseline with one additional loading event (Fig. 5G). Of final note, this persistence of the chromatin condensation state could be completely abrogated via the inhibition of HDAC (TSA) or EZH2 (GSK343) during the unloaded culture following the cessation of loading (Fig. 5H) This suggests that the ‘mechanical memory’ instilled in MSCs subsequent to mechanical perturbation requires ongoing activation of histone modifiers to maintain the altered chromatin state.

## Discussion

In this work we demonstrated that, in the absence of exogenous differentiation factors, mechanical perturbation of MSCs results in rapid chromatin condensation coincident with upregulation of expression of markers of the fibrochondrogenic phenotype. This load-induced change in expression and chromatin architecture required a patent acto-myosin contractility apparatus, in agreement with recent studies^35^,^36^. Furthermore, load induced chromatin reorganization was abrogated with blockade of the histone methyltransferase EZH2, which promotes chromatin condensation and gene silencing^31^. This suggests that EZH2 serves as a common downstream integrator of loading-induced chromatin condensation in this stem cell population.

Chromatin condensation in MSCs persisted with time after loading in a manner that depended on the length of the original mechanical stimulation, altering the mechanical properties of the nucleus itself. This is consistent with previous reports of fluid shear stress induction of chromatin condensation and hardening of endothelial cell nuclei^8^, as well as reorganization of the nuclear lamina and stiffening of isolated nuclei that were stretched *in vitro*^23^. While the latter suggests that nuclear changes are independent of the cell itself, recent work has implicated acto-myosin contractility in chromatin compaction^35^. Cell contractility can change markedly in mechanically stimulated cells^37^. Here, we show that actomyosin based contractility is required for the persistence of chromatin condensation after short term loading, but that condensation in response to longer term loading is insensitive to changes in contractility. This suggests that differential mechanisms may exist; one that first causes nuclear reorganization (via contractility) and another that emerges to preserve this altered state, depending on the duration of load. Indeed, our finding of mechanosensitive changes in cohesin gene expression support the notion of the development of a ‘permanent’ mechanism for genome stabilization with longer term loading.

Adenosine triphosphate (ATP) is a crucial mediator of many cellular functions, including cellular contractility, chromatin remodeling, cell signaling and differentiation^27^,^38^. Numerous studies have shown that mechanical force applied to cells induces the rapid release of ATP and calcium influx into cells^25^,^27^. In this study, we demonstrate that mechanical loading induces rapid ATP release from MSCs, initiating pathways that contribute to chromatin condensation, particularly in the early phase of the response. ATP release decreased with longer durations of loading, however, likely due to exhaustion of ATP stores and/or the ongoing external hydrolysis and utilization by cells. Both loading and addition of exogenous ATP induced the nuclear localization of YAP, consistent with our recent findings showing that dynamic tensile loading activates this pathway in MSCs^11^ and other work reporting similar findings in myoblasts^39^. Further, we found that blocking purinergic signaling via treatment with extracellular ATP hydrolases or hemi-channel blockers abolished this DL-induced YAP nuclear mobilization. Since the YAP pathway can be regulated by G-protein coupled receptor signaling^40^ and ATP is one of the crucial activators of P2Y receptors (a family of purinergic G protein-coupled receptors)^25^, purinergic signaling may also be necessary for loading induced YAP nuclear mobilization, and this may contribute to chromatin remodeling.

Probing these pathways further, we showed using pharmacological blockade that purinergic signaling was involved in the early signaling response (up to 600 seconds), but not in the response to more sustained (3 hours) dynamic loading. It is possible that this is due to differences in ATP release, which are dependent on the duration of loading. Conversely, when calcium in the extracellular media and calcium-responsive signaling elements (such as calmodulin and calcineurin) in the cell were blocked, the response to DL at all time points was completely eliminated. Likewise, when the PIEZO family of mechano-activated calcium channels was blocked, both short term and long term DL-induced chromatin remodeling was eliminated^41^,^42^. This suggests that both the early and long term response to mechanical perturbation depends on calcium entry, but that only the early response is mediated through ATP-related purinergic signaling.

The finding of differences in early and late mechano-response led to the exploration of additional loading scenarios. Interestingly, we found that increasing the number of loading cycles increased the magnitude of chromatin condensation and upregulated expression of genes associated with chromatin movement and stabilization. In addition, when mechanical perturbation was applied multiple times, the magnitude of chromatin condensation and ECM gene expression increased. This suggests that there exists a reserve capacity for remodeling of nuclear chromatin, where repeated loading events may refine and expand locations of condensation within the genome. This advanced state of chromatin condensation also imparted a degree of permanency to the load conditioned state, where increasing the number of loading cycles sustained the condensed state for a longer period of time after cessation of loading. This implies that a mechanical memory is established in the chromatin architecture with dynamic loading. Moreover, this permanence in chromatin condensation depended on the continued activity of both histone methyltransferases and acetylases after loading had stopped, suggesting that ‘memory’ may be encoded in the altered activities of these histone modifiers. Taken together, this work provides new mechanistic insights into the pathways through which external mechanical perturbation is transmitted to the stem cell nucleus to mediate chromatin remodeling and instill within these cells a memory of previous loading events.

## Materials and Methods

### Preparation of aligned nanofibrous scaffolds

Aligned poly(ε-caprolactone) (PCL) nanofibrous scaffolds (~0.50 mm thick) were fabricated by electrospinning^5^. Briefly, a PCL solution (14.3% wt/vol in 1:1 tetrahydrofuran and N,N-dimethylformamide) was loaded into a 20 ml syringe and extruded at a rate of 2.5 ml/hour through an 18G stainless steel needle charged to +13 kV. Fibers were collected onto a cylindrical mandrel rotating with a surface velocity of 10 m/sec to direct fiber alignment. PCL scaffolds (60 × 5 mm^2^) were hydrated and sterilized in decreasing concentrations of ethanol (100, 70, 50, 30%; 30 minutes/step) followed by incubation in a 20 μg/ml solution of fibronectin for ~12 hours to enhance cell attachment.

### MSC isolation and culture on scaffolds

Mesenchymal stem cells (MSCs) were isolated from juvenile bovine tibiofemoral joints (3–6 months old, Research 87, Inc., Boylston, MA) as in^43^. 2 × 10^5^ cells were seeded onto each side of the scaffold followed by culture in a chemically defined serum free medium (CM)^43^ (high glucose DMEM with 1 ×  penicillin/streptomycin/fungizone, 0.1 μM dexamethasone, 50 μg ml^−1^ ascorbate 2-phosphate, 40 μg ml^−1^ l-proline, 100 μg ml^−1^ sodium pyruvate, 6.25 μg ml^−1^ insulin, 6.25 μg ml^−1^ transferrin, 6.25 ng ml^−1^ selenous acid, 1.25 mg ml^−1^ bovine serum albumin and 5.35 μg ml^−1^ linoleic acid) without any additional growth factors.

### Dynamic mechanical perturbation of MSC-seeded scaffolds

MSC seeded scaffolds were loaded into a custom bioreactor^9^. Dynamic tensile loading (DL) was applied in CM at 1Hz for varying durations (up to 6 hours per day) following 2 days of pre-culture of the scaffold in CM. To investigate the effect of acto-myosin contractility on mechanical signaling to the nucleus, constructs were pretreated with 10 μM of the Rho kinase inhibitor, Y27632 for 1 hour (Y27, 10 μM, EMD Millipore, Bedford, MA). In addition, to understand whether DL regulates chromatin condensation via activation of methyltransferases, cells were pre-treated with an inhibitor of the histone H3K27 methyltransferase EZH2, GSK343 for 2 days before application of DL.

### Calculation of the chromatin condensation parameter (CCP)

To assess chromatin condensation, constructs were incubated in 4% paraformaldehyde for 30 mins at 37 °C to fix cells, followed by washing and permeabilization with 0.05% Triton X-100 in PBS supplemented with 320 mM sucrose and 6mM magnesium chloride. Nuclei were visualized by DAPI (ProLong® Gold anti-fade reagent with DAPI, P36935, Molecular Probes®, Grand Island, NY) staining and scanned across their mid-section using a confocal microscope (Zeiss, LSM 510, Jena, Germany). To calculate the chromatin condensation parameter (CCP), a gradient-based Sobel edge detection algorithm was employed using MATLAB to measure the edge density for individual nuclei^30^.

### Analysis of gene expression by real time PCR

Total RNA was isolated from constructs using TRIZOL, and quantified using a Nanodrop spectrometer (ND-1000, Nanodrop Technologies, Wilmington, DE, USA). cDNA was synthesized using the SuperScript First Strand Synthesis kit (Invitrogen, Life Technologies, Carlsbad, CA, USA). Amplification was performed using an Applied Biosystems Step One Plus real-time PCR system, with Fast SYBR Green Reaction Mix (#4385617, Applied Biosystems, Foster City, CA, USA). Expression of TGF-β (CACGTGGAGCTGATCCAGAA and ACGTCAAAGGACAGCCACTC), aggrecan (AGG, CTGAACGACAAGACCATCGA and TGGCAAAGAAGTTGTCAGGC), CTCF (TCGACCTGAATGATGGCTGTT and CCCACCACCTGCCAAGAA), and SMC1A (TCCCCCCCTGACAAGTTGT and CCCTACTTGGATGGCATAAAGTACA) were determined and normalized to the housekeeping gene glyceraldehyde-3-phosphate dehydrogenase (GAPDH, ATCAAGAAGGTGGTGAAGCAGG and TGAGTGTCGCTGTTGAAGTCG).

### Assessment of permanency of condensation and nuclear mechanics

To evaluate permanency of load-induced chromatin condensation after the cessation of loading, constructs were dynamically loaded (3%, 1 Hz) for different durations (150 s, 600 s, 1 h or 3 h) followed by culture for an additional 18 hours. To investigate the effect of acto-myosin contractility on the permanency of condensation, another set of constructs was pretreated with Y27632 after loading.

To quantify changes in nuclear mechanics, the nuclear aspect ratio (NAR = a/b, Fig. 2C) was assessed under baseline conditions and after applying 9 and 15% grip-to-grip static strain to constructs. Nuclei were stained with DAPI and cell-seeded scaffolds were placed on a custom device designed to permit visualization on an epi-fluorescent inverted microscope during stretch. All images were captured on an inverted fluorescent microscope (Nikon T30, Nikon Instruments, Melville, NY) equipped with a CCD camera. At each strain level, NAR was calculated using a custom MATLAB code. Changes in NAR were tracked for individual MSC nuclei at each strain step as in^11^.

To confirm whether chromatin condensation increases nuclear stiffness, MSCs were treated with exogenous divalent cations (MgCl_2_ and CaCl_2_, 2 mM each, Sigma-Aldrich) in CM for 30 min^44^. CCP and NAR were determined after treatment as described above. In addition, MSC nuclear stiffness was determined by atomic force microscopy (AFM, DAFM-2X, Veeco, Woodbury, NY). For this, MSCs were removed from scaffolds by trypsinization and re-plated onto tissue culture plastic for 24 hours. MSCs were treated with divalent cations for 30 min and the cells were probed using a silicon nitride probe with a pyramidal tip (spring constant of 0.06 N/m, DNP, Veeco). Peri-nuclear stiffness was determined from the force-indentation curve using a Hertz model^45^.

### Analysis and pharmacologic blockade of purinergic signaling in MSCs

To determine the effector agent in load-conditioned media, DL-conditioned medium was size fractionated using an Amicon Ultra-15 Centrifugal Filter Unit (3,000 × g for 20 mins at °C, 3 kDa MWCO, UFC9000308, EMD Millipore). The filtered medium (containing molecules <3 kDa) or the supernatant (molecules >3 kDa) was added to naïve (unloaded) MSC-seeded constructs. After 30 min, these constructs were fixed and CCP was determined. After establishing that the molecular factor was <3 kDa, we next determined whether ATP was released from cells by collecting DL-conditioned media (3%, 1 Hz, 600 s or 3 h). In some cases, the pre-conditioned media was treated with Apyrase (an ATP diphosphohydrolase, AP, 5U/ml) for 30 minutes. The conditioned medium (treated with Apyrase or not) was then added to naïve (unloaded) MSC-seeded constructs. After 30 min, the constructs were fixed and CCP was determined (Fig. 3A). To measure the concentration of ATP in conditioned media, a commercial luciferin/luciferase based ATP Assay Kit was used (KA1661, Abnova, Taipei City, Taiwan), according to manufacturer’s instructions. To determine the degradation rate of ATP in media, MSC seeded scaffolds were dynamically loaded for 600s and then incubated at 37 °C for up to 3 hours. To determine the role of cell utilization in this process, the culture medium was also collected after loading and added to a new culture dish (without cells) and maintained at 37 °C for the same time period.

In some experiments, MSC seeded scaffolds were incubated with various inhibitors to dissect the roles of purinergic signaling pathway mediators. For example, Apyrase (5U, A6535 Sigma-Aldrich, St. Louis, MO)^28^, Flufenamic acid (FFA, 500 μM, F9005, Sigma-Aldrich)^46^, or Oligomycin (Oligo, 10 μg/ml, O4876, Sigma-Aldrich)^34^ were applied for 30 minutes prior to application of dynamic loading and during loading, after which, constructs were fixed and CCP quantified. In a dose response study, ATP (0 ~ 5 mM, R0441, Thermo Scientific), UTP (100 μM, known as a P2 receptor agonist, Sigma-Aldrich), or BzATP (100 μM, known as a P2X activator) was provided to MSCs seeded on scaffolds for 30 minutes prior to fixation and quantification of CCP values.

### Analysis of YAP nuclear localization

YAP (a transcriptional regulator) nuclear localization was evaluated with the addition of ATP (1 mM, 30 min) or with dynamic loading (3%, 1 Hz, 30 min). After each treatment, MSCs were fixed in 4% paraformaldehyde for 30 min at 37 °C and then permeabilized with 0.05% Triton X-100 in PBS supplemented with 320mM sucrose and 6 mM magnesium chloride. Samples were then incubated with anti-YAP antibody in 1% BSA in PBS (1:200, sc-101199, Santa Cruz Bio, Dallas, Texas) for 90 min at room temperature (RT) followed by incubation with Alexa-Fluor 546 goat anti-mouse secondary (1:200, A-11030, Molecular Probes, Grand Island, NY) at RT. For actin staining, samples were incubated for 60 min with Alexa-Fluor 488 (1:1000, A12379, Molecular Probes, Grand Island, NY). Nuclei were stained with 4′, 6-diamidino-2-phenylindole (DAPI, ProLong® Gold antifade reagent with DAPI P36935, Molecular Probes®, Grand Island, NY). Images were taken using a fluorescence microscope with 100 ×  objective (Zeiss Axioplan-2 fluorescent microscope, Jena, Germany). Average YAP staining intensity and localization were quantified using ImageJ (with nuclear staining intensity normalized to cytoplasmic staining) as in^11^.

### Visualization and pharmacological blockade of calcium signaling in MSCs

To determine changes in intracellular Ca^2+^ with application of dynamic loading or addition of exogenous ATP, MSCs on scaffolds were loaded with the fluorescent calcium indicator Cal-520TM AM (15 μM, AAT Bioquest, Sunnyvale, CA) for 1 h at 37 °C. Constructs were placed in a custom micro-tensile device^47^ and preloaded to 30 mN. After preload, images were obtained using a confocal microscope (LSM 5 LIVE; Carl Zeiss, Jena, Germany) every 4 s for 10 min (0.25 Hz scanning frequency). The same process was repeated after ATP addition or application of dynamic loading. A custom MATLAB (The Mathworks Inc., Natick, MA, USA) program was used to analyze [Ca^2+^]_i_ oscillations (amplitude, time between peaks, and number of peaks in ten minutes) as in^47^.

To investigate the role of calcium signaling in load-induced chromatin condensation, constructs were incubated for 30 min prior to the application of loading with various small molecular modulators including BAPTA (50 μM, A4926, Sigma)^34^, Ruthenium Red (RR, R2751, 10 μM, Sigma)^48^, GSK205 (G205, 10 μM, 616522, EMD Millipore)^49^, Thapsigargin (TG, 5 μM, T9033, Sigma)^50^, GdCl_3_ (GC, 100 μM, 439770, Sigma)^34^, Verapamil (VP, 10 μM, V4629, Sigma)^28^, PPADS (100 μM, ab120009, Abcam)^34^, EGTA (1 mM, E3889, Sigma)^48^, CALP2 (CALP, 20 μM, 2319, TOCRIS)^51^ or GsMTx-4 (3 μM, ab141871, Abcam)^52^. BAPTA-AM (50 μM, A1076, Sigma)^53^ was added to constructs overnight. KN-62 (KN62, 10 μM, 1277, TOCRIS)^54^ and cyclosporin A (CYSP, 5 μM, tlrl-cyca, InvivoGen)^53^ were added for 1 hour before loading. To determine whether the pharmacologic inhibitors altered chromatin condensation or nuclear response to stretch, baseline CCP was monitored with each treatment and the change in nuclear aspect ratio (NAR) was assessed at 9% and 15% static stretch as in^11^.

### Assessment of mechanical memory after loading

To determine the factors involved in mechanical memory, another set of constructs was dynamically loaded for 3 hours/day (3%, 1Hz), returned to free swelling culture for 48 hours, and then subjected to a further round of loading for 3 hours (3%, 1 Hz) following free swelling culture for 48 hours. CCP, nuclear deformation with static stretch, and expression of AGG, SMC1A, and CTCF were quantified at each time point. For each condition, the nuclear deformation index (NDI) was calculated by normalizing nuclear deformation at each strain level to the mean deformation observed for the control group at that same strain level, as in^11^.

To explore the impact of repeated mechanical loading, MSC-seeded constructs were dynamically loaded (3%, 1 Hz, 6hour/day) for 1 day (DL × 1), 3 days (DL × 3) or 7 days (DL × 7), after which they were returned to free swelling culture for an additional 5 days. Changes in AGG gene expression were determined at set time points. To determine the roles of EZH2 and HDAC during this process, GSK343 or TSA were added to the mechanically pre-conditioned MSCs after the cessation of loading. To determine whether previous loading events influenced changes in CCP with an additional loading event, another set of constructs was dynamically loaded (3%, 1 Hz, 6hour/day) for 1 day (DL × 1) or 7 days (DL × 7), returned to free swelling culture for 5 days, and then subjected to another round of loading (6 hours). At set time points after cessation of loading, CCP was measured along with changes in gene expression by real time RT-PCR.

### Statistical analyses

Statistical analysis was performed using ANOVA with Fisher’s LSD post hoc testing (SYSTAT v.10.2, Point Richmond, CA). Results are expressed as mean ± the standard error of the mean (s.e.m.) or standard deviation (s.d.), as indicated in the figure legends. Differences were considered statistically significant at p < 0.05. Sample and replicate numbers are provided in the figure legends.

## Additional Information

**How to cite this article**: Heo, S.-J. *et al.* Biophysical Regulation of Chromatin Architecture Instills a Mechanical Memory in Mesenchymal Stem Cells. *Sci. Rep.* **5**, 16895; doi: 10.1038/srep16895 (2015).

## Supplementary Material

Supplementary Figures
