# Supplementary material for: Biophysical Regulation of Chromatin Architecture Instills a Mechanical Memory in Mesenchymal Stem Cells
Source: Sci Rep. 2015 Nov 23;5:16895. doi: 10.1038/srep16895 (PMC4655352; doi:10.1038/srep16895)

Supplementary Information for:

Biophysical Regulation of Chromatin Architecture Instills a Mechanical Memory in Mesenchymal Stem Cells

Su-Jin Heo[1,2], Stephen D. Thorpe[3], Tristan P. Driscoll[1,2,4], Randall L. Duncan[5], David A. Lee[3], and Robert L. Mauck[1,2,4]*

[1]McKay Orthopaedic Research Laboratory, Department of Orthopaedic Surgery, Perelman School of Medicine, University of Pennsylvania, Philadelphia, PA, USA

[2]Department of Bioengineering, School of Engineering and Applied Science, University of Pennsylvania, Philadelphia, PA, USA

[3]Institute of Bioengineering, School of Engineering and Materials Science, Queen Mary University of London, London, UK

[4]Translational Musculoskeletal Research Center, Philadelphia VA Medical Center, Philadelphia, PA, USA

[5]Department of Biological Sciences, University of Delaware, Newark, DE, USA

***Address for Correspondence**:
Robert L. Mauck, Ph.D.
Associate Professor of Orthopaedic Surgery and Bioengineering
McKay Orthopaedic Research Laboratory
Department of Orthopaedic Surgery
Perelman School of Medicine
University of Pennsylvania
36th Street and Hamilton Walk
Philadelphia, PA 19104
Phone: (215) 898-3294
Fax: (215) 573-2133
Email: lemauck@mail.med.upenn.edu

SUPPLEMENTAL FIGURE LEGENDS

Supplemental Figure 1. Persistence of chromatin condensation with short term DL (600s, 1Hz) depends on the magnitude of applied strain (red line: unstrained CM control, DL: 600s, 1Hz, n = ~20, *: $p<0.05$ vs. CM control, +: $p<0.05$ vs. 3%, α: $p<0.05$ vs. 0s, mean ± s.e.m.).

Supplemental Figure 2. Chromatin condensation correlates with an increase in nuclear mechanics and a decrease in in situ nuclear deformation. (**A**) Treatment with $MgCl_2$+$CaCl_2$ for 30 minutes increases chromatin condensation (top) and the number of visible edges in DAPI stained nuclei (bottom, bar = 3 μm). (**B**) Increased CCP with addition of $MgCl_2$+$CaCl_2$ (n = ~ 20 cells, *: $p<0.05$ vs. 0 mM, +: $p<0.05$ vs. 10 mM, mean ± s.e.m.). (**C**) Nuclear aspect ratio (NAR) as a function of treatment and with applied scaffold stretch (n = ~ 45, *: $p<0.05$ vs. 0%, +: $p<0.05$ vs. 9%, ×: $p<0.05$ vs. 0 mM, mean ± s.e.m.). (**D**) Peri-nuclear stiffness measured by atomic force microscopy (AFM) increases with an increase in chromatin condensation in response to $MgCl_2$+$CaCl_2$ treatment (n = 10, *: $p<0.05$ vs. 0 mM, mean ± s.d.).

Supplemental Figure 3. Normalized CCP (relative to unloaded MSCs) after treatment for 30 minutes with complete or size fractionated DL-conditioned media (red line: unloaded CM control, n = ~20, *: $p<0.05$ vs. CM control, mean ± s.e.m.).

Supplemental Figure 4. (**A**) CCP increases with the addition of exogenous ATP (n = ~20, *: $p<0.05$ vs. 0 mM, mean ± s.e.m.). (**B**) UTP addition increased CCP, whereas BzATP added at the same concentration had no effect on CCP (n = ~20, *: $p<0.05$ vs. CM control, mean ± s.e.m.).

Supplemental Figure 5. Degradation of ATP in DL-conditioned media. ATP released from MSCs after 600s of DL gradually degraded, and did so at a faster rate when cells were present (37°C, n = ~ 3, *: $p<0.05$ vs. without cells, +: $p<0.05$ vs. 30m, α: $p<0.05$ vs. 1h, β: $p<0.05$ vs. 2h, normalized to ATP levels after 600s DL, mean ± s.d.).

Supplemental Figure 6. (**A-C**) Representative images of YAP staining with treatment; (**A**): CM control, (**B**): 1mM ATP for 30 min, (**C**): 3% DL at 1Hz for 30 min (red: YAP, green: actin, blue: nucleus). (**D**) Nuclear to cytoplasmic YAP ratio with the addition of ATP or application of DL for 30 min normalized to CM control (n = ~15, *: $p<0.05$ vs. CM control, mean ± s.d.). (**E**) Ratio of nuclear to cytoplasmic YAP with the application of DL for 30 min under control conditions or with apyrase (AP, 5U) or flufenamic acid (FFA: a hemichannel blocker) added to the media during loading. Data normalized to unloaded CM control (red line)(n = ~15, *: $p<0.05$ vs. CM control, mean ± s.d.).

Supplemental Figure 7. Alterations in CCP with short and long term dynamic loading and pre-treatment with various inhibitors; (**A**): EGTA (a calcium chelator), (**B**): CALP2 (CALP, an antagonist of Calmodulin), (**C**): Cyclosporine A (CYSP, a Calcineurin inhibitor), (**D**): BAPTA-AM (BATAM, a calcium chelator), (**E**): Ruthenium red (RR, a TRPV4 inhibitor), (**F**): GSK205 (G205,

a TRPV4 antagonist), (**G**): GsMTx4 (GMT, a PIEZO ion channel inhibitor), (**H**): $GdCl_3$ (GC, a stretch-activated channel inhibitor), (**I**): PPADS (a P2 receptor antagonist). (DL: dynamic loading, red line: CM control, green line: DL 600s, blue line: DL 3h, n = ~20 per condition, *: p<0.05 vs. CM control, mean ± s.e.m.).

Supplemental Figure 8. Control studies showing no marked changes in (**A**) the baseline CCP (n= ~20) with the addition of pharmacological inhibitors for 600s or 3 hrs in unloaded conditions. (**B**) Nuclear deformation in MSCs subjected to static stretch with the addition of pharmacological inhibitors (n = ~50, CALP: CALP2, TG: thapsigargin, GC: $GdCl_3$, GSK: GSK205, *: p<0.05 vs. 0%, +: p<0.05 vs. 9% scaffold stretch, mean ± s.e.m.).

Supplemental Figure 9. TGF-β (**A**), SMC1A (**B**) and CTCF (**C**) gene expression normalized to CM control (red line: CM control, n = 9, from 3 replicates, *: p<0.05 vs. CM control, +: p<0.05 vs. a, ‡: p<0.05 vs. b, α: p<0.05 vs. c, mean ± s.e.m.).

Supplemental Figure 10. Change in aggrecan expression (AGG) as a function of the number of DL events and time after cessation of loading (n = ~ 3, *: p<0.05 vs. CM control (red line), +: p<0.05 vs. day 0, ‡: p<0.05 vs. day 3, mean ± s.d.).

Supplemental Figure 1

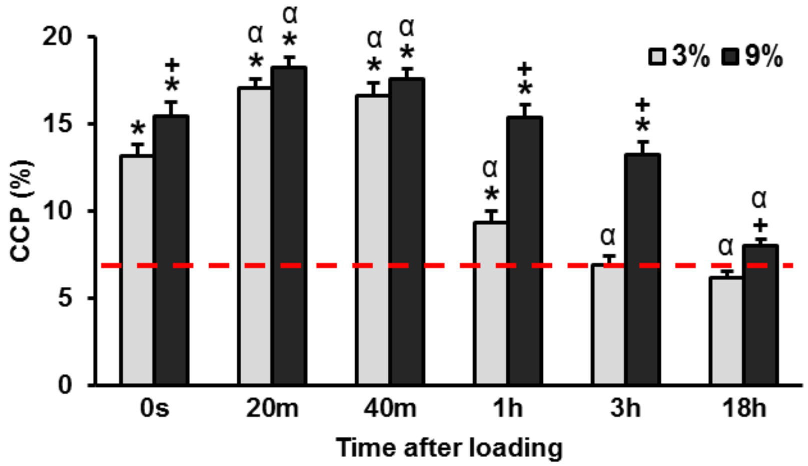

Supplemental Figure 2

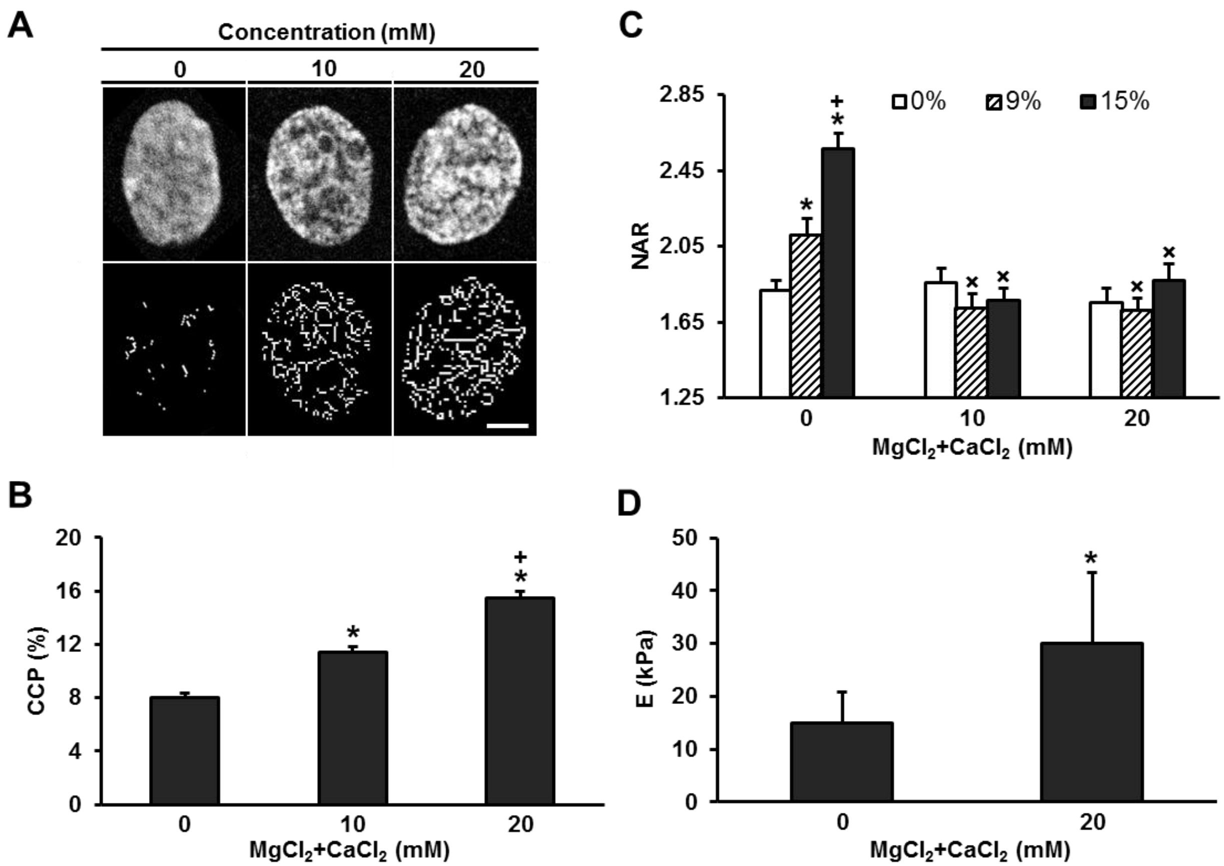

Supplemental Figure 3

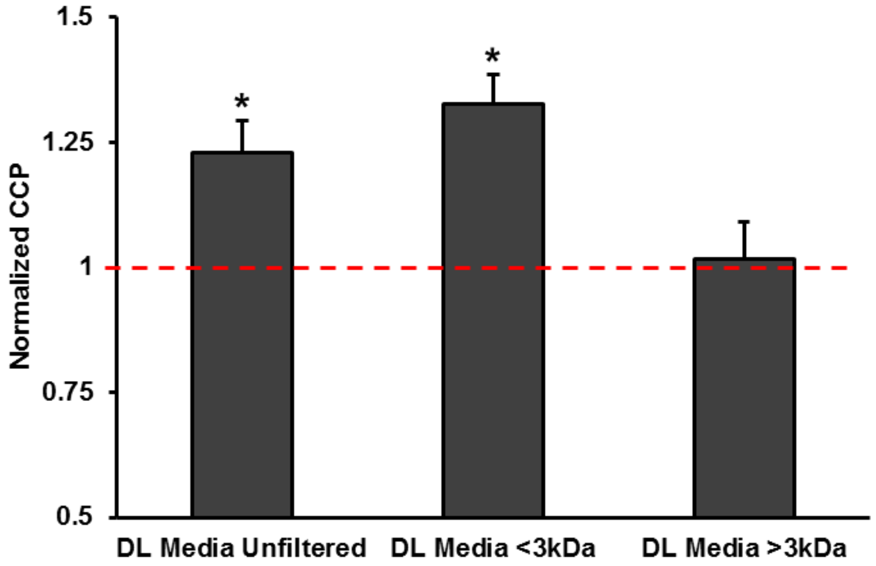

Supplemental Figure 4

.

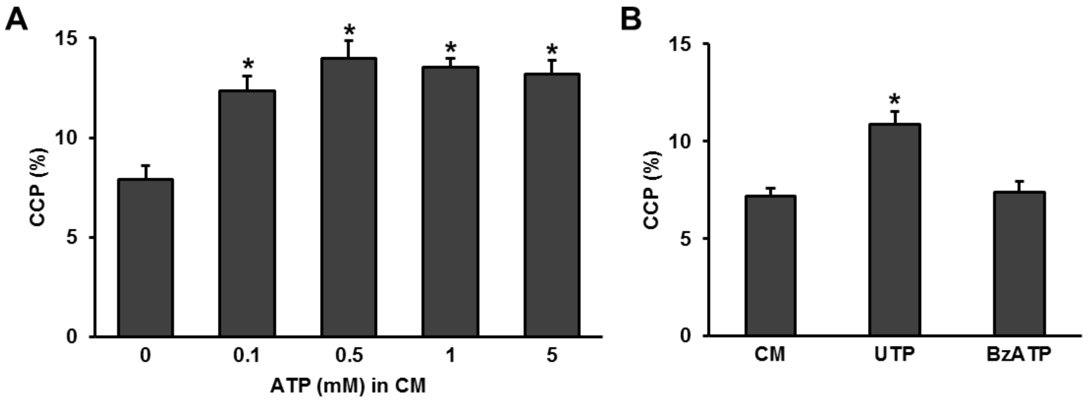

Supplemental Figure 5

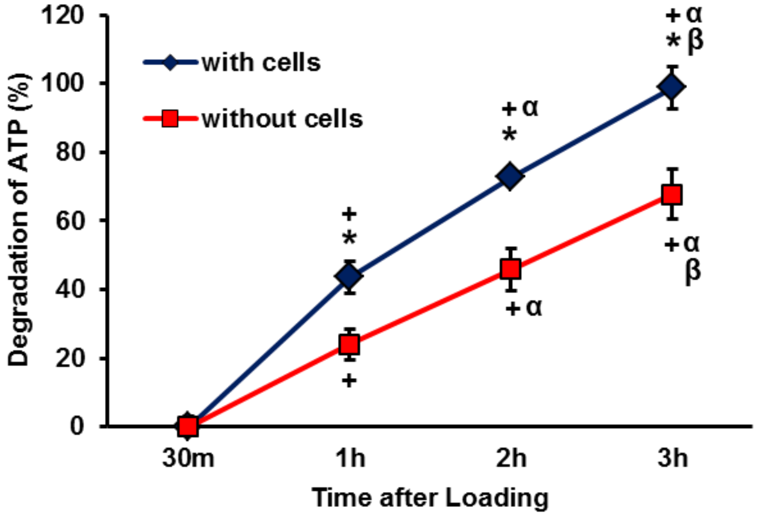

Supplemental Figure 6

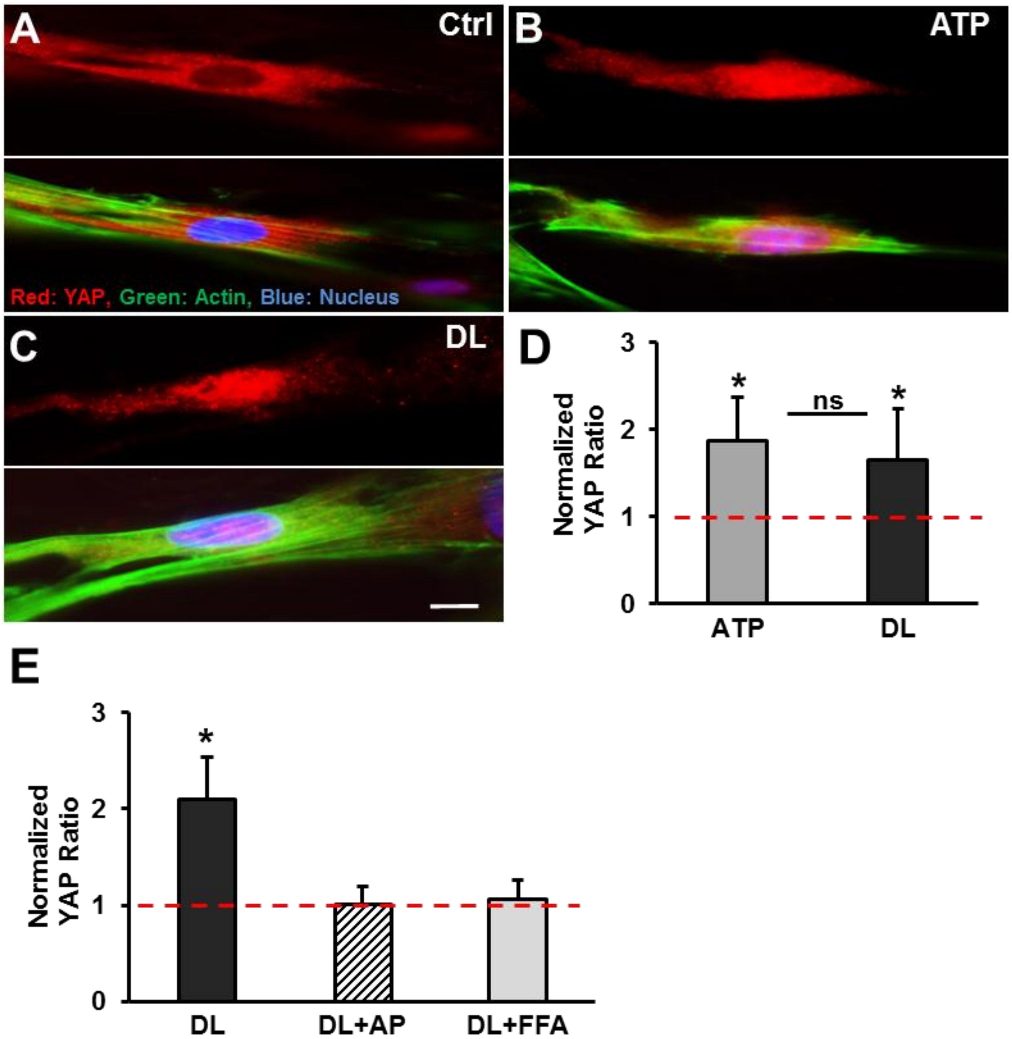

Supplemental Figure 7

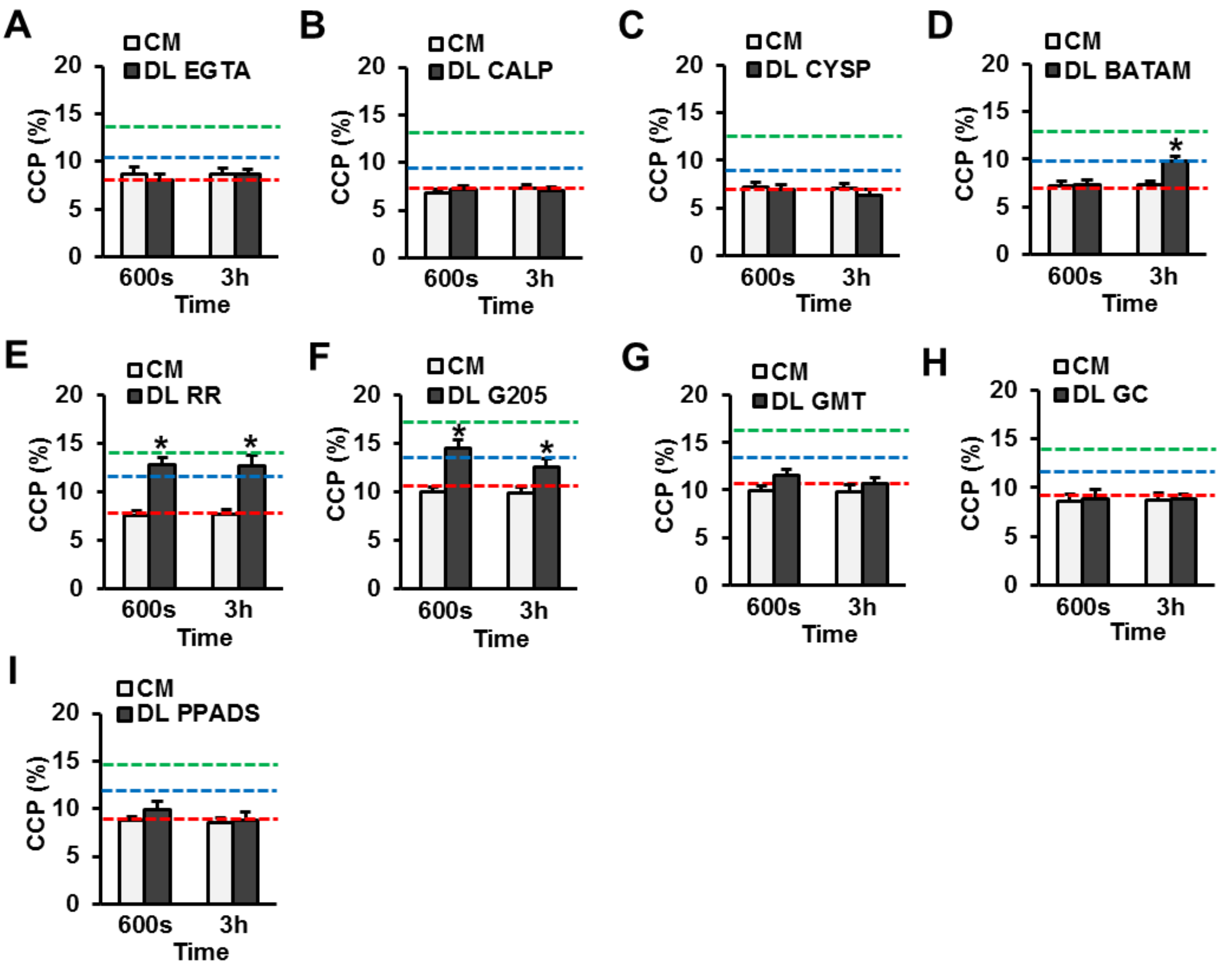

Supplemental Figure 8

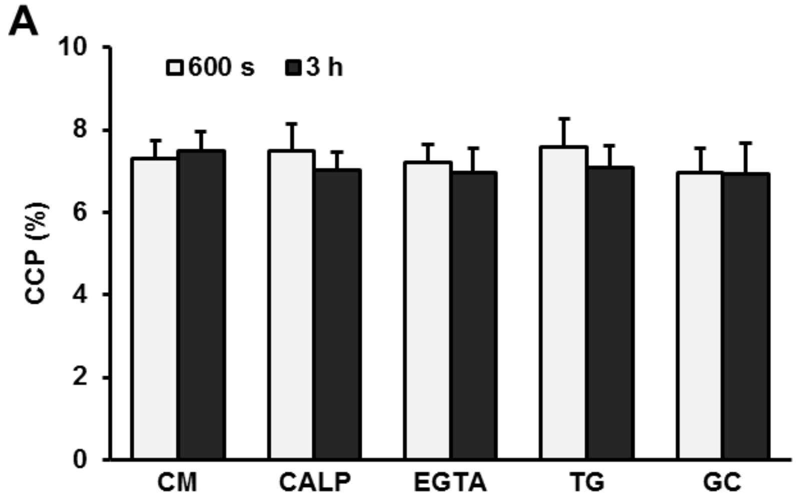

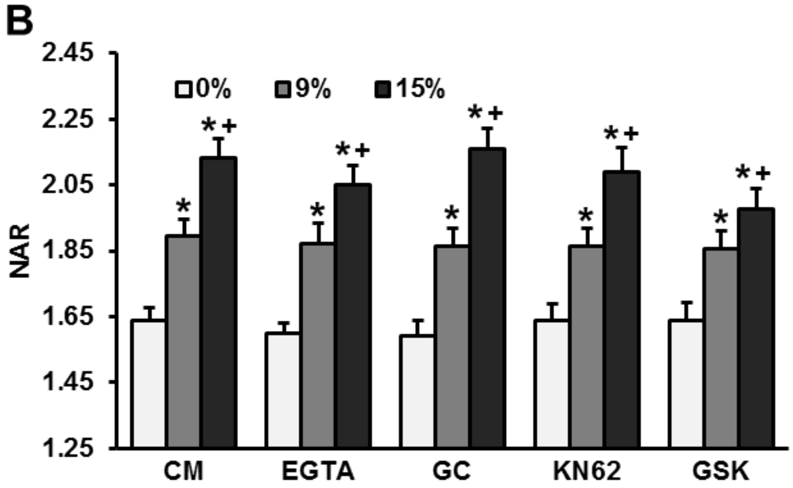

Supplemental Figure 9

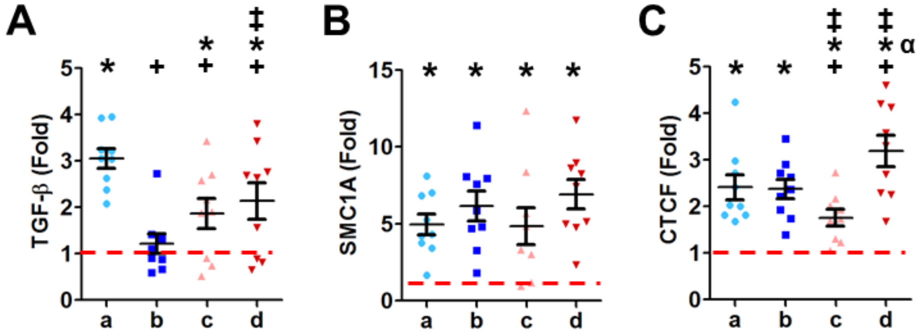

Supplemental Figure 10

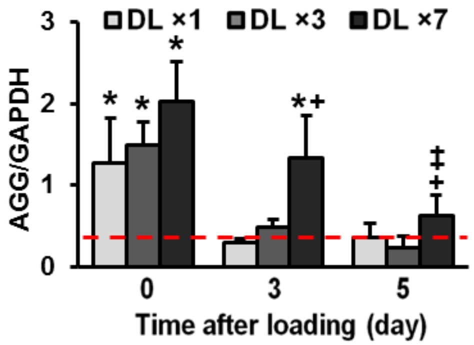

Supplement: Supplementary Figures [file srep16895-s1.pdf]
